# Supplementary material for: Distribution of Yeast Species and Risk Factors of Oral Colonization after Oral-Care Education among the Residents of Nursing Homes
Source: J Fungi (Basel). 2022 Mar 17;8(3):310. doi: 10.3390/jof8030310 (PMC8953697; doi:10.3390/jof8030310)
Supplement: Supplementary file 1 [file jof-08-00310-s001.zip › jof-1609277-supplementary.pdf]

Journal of Fungi

MDPI

Table S1. Distribution of yeast species

Participant	No. of spp.	*C. albicans*	*C. glabrata*	*C. parapsilosis*	*C. tropicalis*	*C. pararugosa*	Others
TCH-029-27	5	1	0	0	0	1	*C. krusei, C. orthopsilosis, Lodderomyces elongisporus*
GLS-011-144	5	1	0	1	0	0	*C. intermedia, C. lusitaniae, Pichia aff. fermentans*
GLS-019-167	4	0	0	1	0	1	*Exophiala dermatitidis, Trichosporon mucoides*
TCH-011-118	4	1	0	0	1	1	*Saccharomyces cerevisiae*
FSC-021-90	4	1	0	1	0	0	*C. guilliermondii, C. orthopsilosis*
HWA-012-21	4	1	0	1	0	1	*C. intermedia*
HWA-017-63	4	1	1	0	0	0	*C. metapsilosis, Magnusiomyces capitatus*
FSC-049-93	4	1	1	0	1	0	*Saccharomyces cerevisiae*
GFC-014-154	4	1	1	0	1	0	*C. orthopsilosis*
FSC-001-33	4	1	1	1	0	0	*Candida guilliermondii*
HWA-015-127	3	0	0	0	0	0	*C. intermedia,*
GLS-017-136	3	0	0	1	0	1	*Trichosporon asahii*
YEOo-002-89	3	1	0	0	0	0	*C. guilliermondii,Magnusiomyces capitatus*
FAM-007-168	3	1	0	1	0	0	*C. norvegensis*
FSC-041-126	3	1	0	1	0	0	*Saccharomyces cerevisiae*
FSC-008-46	3	1	0	1	0	0	*C. fermentati*
FSC-004-42	3	1	0	1	0	0	*C. guilliermondii*
TCH-013-104	3	1	0	1	0	0	*Trichosporon asahii*
FAM-001-73	3	1	0	1	0	1	

WES-001-2	3	1	0	1	1	0	
FSC5-039-114	3	1	1	0	0	0	*Trichosporon asahii*
FSC-029-68	3	1	1	0	0	0	*Rhodotorula minuta*
GLS5-010-66	3	1	1	0	0	0	*Candida ethanolica*
FAM-011-34	3	1	1	0	1	0	
FSC5-025-11	3	1	1	0	1	0	
FSC5-016-50	3	1	1	0	1	0	
GLS-008-15	3	1	1	0	1	0	
GLS-007-67	3	1	1	0	1	0	
HWA-002-13	3	1	1	0	1	0	
TCH-010-78	3	1	1	0	1	0	
WES-013-96	3	1	1	0	1	0	
WES-018-40	3	1	1	0	1	0	
WES-020-75	3	1	1	0	1	0	
YEO5-006-108	3	1	1	0	1	0	
TCH-014-117	3	1	1	1	0	0	
HWA-007-56	2	0	1	0	0	0	*Candida dubliniensis*
TCH5-016-115	2	0	1	0	0	0	*Candida metapsilosis*
FSC-042-155	2	1	0	0	0	0	*Saccharomyces cerevisiae*
HOP-008-148	2	1	0	0	0	0	*Trichosporon asahii*
HWA-014-138	2	1	0	0	0	0	*Exophiala dermatitidis*
HWA-001-157	2	1	0	0	0	0	*Candida guilliermondii*
YEO-008-35	2	1	0	0	0	0	*Candida metapsilosis*
GFC-015-163	2	1	0	0	1	0	
HOP-001-101	2	1	0	0	1	0	
TCH5-043-113	2	1	0	0	1	0	
GLS-014-110	2	1	0	1	0	0	

TCH-021-6	2	1	0	1	0	0	
FSC-026-76	2	1	1	0	0	0	
FSC5-023-38	2	1	1	0	0	0	
FSC-033-137	2	1	1	0	0	0	
FSC-018-57	2	1	1	0	0	0	
GLS-003-102	2	1	1	0	0	0	
GLS-015-24	2	1	1	0	0	0	
GLS-018-16	2	1	1	0	0	0	
GLS-003-128	2	1	1	0	0	0	
HOP-003-7	2	1	1	0	0	0	
HOP-005-111	2	1	1	0	0	0	
HOP-006-29	2	1	1	0	0	0	
HOP-010-121	2	1	1	0	0	0	
HWA-020-39	2	1	1	0	0	0	
HWA-009-53	2	1	1	0	0	0	
HWA-006-109	2	1	1	0	0	0	
TCH-037-150	2	1	1	0	0	0	
TCH5-009-25	2	1	1	0	0	0	
TCH-024-62	2	1	1	0	0	0	
WES-016-92	2	1	1	0	0	0	
WES-019-20	2	1	1	0	0	0	
TCH-012-149	1	0	0	0	0	0	*C. utilis*
WES-040-44	1	0	0	0	0	0	*Candida dubliniensis*
YEO-009-141	1	0	0	0	0	0	*Candida dubliniensis*
YEO-003-52	1	0	0	0	0	0	*Candida dubliniensis*
HWA-037-123	1	0	0	0	0	1	
GFC-022-98	1	0	0	1	0	0	

GFC-004-94	1	0	0	1	0	0
HOP-007-145	1	0	0	1	0	0
TCH-006-4	1	0	0	1	0	0
FAM-006-146	1	1	0	0	0	0
FAM-004-69	1	1	0	0	0	0
FAM-002-125	1	1	0	0	0	0
FAM-008-70	1	1	0	0	0	0
FSC-031-14	1	1	0	0	0	0
FSC-030-91	1	1	0	0	0	0
FSC-027-51	1	1	0	0	0	0
FSC-019-12	1	1	0	0	0	0
FSC-002-3	1	1	0	0	0	0
FSC-012-41	1	1	0	0	0	0
FSC-032-105	1	1	0	0	0	0
FSC-007-122	1	1	0	0	0	0
FSC-044-82	1	1	0	0	0	0
FSC-014-18	1	1	0	0	0	0
FSC-013-71	1	1	0	0	0	0
GFC5-002-95	1	1	0	0	0	0
GFC-017-119	1	1	0	0	0	0
GLS5-004-134	1	1	0	0	0	0
GLS-006-81	1	1	0	0	0	0
GLS5-012-165	1	1	0	0	0	0
GLS-007-22	1	1	0	0	0	0
GLS-001-97	1	1	0	0	0	0
GLS-002-99	1	1	0	0	0	0
HWA-013-156	1	1	0	0	0	0

TCH-004-85	1	1	0	0	0	0
TCH-049-133	1	1	0	0	0	0
TCH-015-143	1	1	0	0	0	0
TCH-027-48	1	1	0	0	0	0
TCH-046-124	1	1	0	0	0	0
TCH-007-17	1	1	0	0	0	0
TCH-035-107	1	1	0	0	0	0
TCH-019-140	1	1	0	0	0	0
WES-004-120	1	1	0	0	0	0
WES-017-55	1	1	0	0	0	0
WES-007-74	1	1	0	0	0	0
WES-038-45	1	1	0	0	0	0
WES-002-130	1	1	0	0	0	0
WES5-021-54	1	1	0	0	0	0
WES-011-86	1	1	0	0	0	0
WES-023-164	1	1	0	0	0	0
YEO-007-58	1	1	0	0	0	0

No, number.

Table S2. Distribution of species causing multiple species colonization

Total spp No	No. residents	*C. albicans*	*C. glabrata*	*C. parapsilosis*	*C. tropicalis*	*C. pararugosa*	Others
5	1	1	0	1	0	0	*C. intermedia, C. lusitaniae, Pichia aff. fermentans*
5	1	1	0	0	0	1	*C. krusei, C. orthopsilosis, Lodderomyces elongisporus*
4	1	1	1	1	0	0	*C. guilliermondii*
4	1	1	1	0	1	0	*Saccharomyces cerevisiae*
4	1	1	1	0	1	0	*C. orthopsilosis*
4	1	1	1	0	0	0	*C. metapsilosis, Magnusiomyces capitatus*
4	1	1	0	1	0	1	*C. intermedia*
4	1	1	0	1	0	0	*C. guilliermondii, C. orthopsilosis*
4	1	1	0	0	1	1	*Saccharomyces cerevisiae*
4	1	0	0	1	0	1	*Exophiala dermatitidis, Trichosporon mucoides*
3	11	1	1	0	1	0	
3	1	1	1	1	0	0	
3	1	1	1	0	0	0	*Trichosporon asahii*
3	1	1	1	0	0	0	*Rhodotorula minuta*
3	1	1	1	0	0	0	*C. ethanolica*
3	1	1	0	1	1	0	
3	1	1	0	1	0	1	

3	1	1	0	1	0	0	*C. norvegensis*
3	1	1	0	1	0	0	*Saccharomyces cerevisiae*
3	1	1	0	1	0	0	*C. fermentati*
3	1	1	0	1	0	0	*C. guilliermondii*
3	1	1	0	1	0	0	*Trichosporon asahii*
3	1	1	0	0	0	0	*C. guilliermondii, Magnusiomyces capitatus*
3	1	0	0	1	0	1	*Trichosporon asahii*
3	1	0	0	0	0	0	*C. intermedia,*
2	20	1	1	0	0	0	
2	3	1	0	0	1	0	
2	2	1	0	1	0	0	
2	1	1	0	0	0	0	*Saccharomyces cerevisiae*
2	1	1	0	0	0	0	*Trichosporon asahii*
2	1	1	0	0	0	0	*Exophiala dermatitidis*
2	1	1	0	0	0	0	*C. guilliermondii*
2	1	1	0	0	0	0	*C. metapsilosis*
2	1	0	1	0	0	0	*C. dubliniensis*
2	1	0	1	0	0	0	*C. metapsilosis*

No, number.
